# Biomimetic synthesis of nanostructured WO_3_ · H_2_O particles and subsequent thermal conversion to WO_3_

**DOI:** 10.1098/rsos.182137

**Published:** 2019-06-12

**Authors:** Hiroaki Uchiyama, Shouta Mizuguchi, Shiho Hirano

**Affiliations:** Department of Chemistry and Materials Engineering, Kansai University, 3-3-35 Yamate-cho, Suita 564-8680, Japan

**Keywords:** WO_3_, nanoparticles, biomimetic synthesis, aqueous solution process

## Abstract

Nanostructured tungsten oxide (WO_3_) particles were prepared in aqueous solution by mimicking biomineralization. Precursor WO_3_ · H_2_O particles were generated by ageing a 60°C (NH_4_)_10_W_12_O_41_ · 5H_2_O solution containing gelatin. This was followed by heating to 600°C in air for thermal conversion to WO_3_. The addition of gelatin led to the formation of layered structures consisting of WO_3_ · H_2_O platy particles, which contained segmented, block-like nanoscale units. The macroscopic layered structure was preserved after thermal conversion to WO_3_, while the morphology of the block-like units changed to orthogonally crossed nanorods.

## 1. Introduction

Tungsten oxide (WO_3_) has been used in various devices such as photoelectrodes [1–3], electrochromic materials [1,4,5] and gas sensors [6–8]. Device performance is affected by crystallite size and shape and the morphology of the secondary particles. Thus, nanostructural control of WO_3_ crystals is important for practical applications.

Recently, various nanostructured inorganic materials have been prepared by novel solution techniques that mimicked biomineralization. Natural biominerals such as nacres, sea urchin spines and eggshells consist of oriented inorganic units less than 50 nm in size, and nanoscale spaces containing biological polymers like chitin, chitosan and gelatin [9–17]. Such highly ordered nanostructures are formed by self-assembly and self-organization via interactions between the inorganic crystals and the biological polymers. The polymers contain many polar amino acids [18] that adsorb on the surfaces of the inorganic crystals, creating hierarchical structures consisting of nanoscale crystallites. Biomimetic structures have been widely made from nanoscale inorganic units and biological polymers [11–13,18–25]. Many works about the biomimetic synthesis of functional metal oxide materials mainly focused on the similarity in the resultant nanostructure between the products and the real biominerals, and the resultant device performance. On the other hand, we have focused on ‘biomimetic synthetic route’ and attempted to construct novel aqueous techniques for making nanostructured materials. We think that the key factors of biomineralization are (i) the interaction between inorganic crystals and biological polymers, and (ii) the multistep synthetic procedure via metastable phases as the precursor materials, and have suggested new approaches containing one or both factors for making hierarchical structures consisting of oriented nanocrystallites like biominerals. Cocoon-like CeCO_3_OH particles consisting of nanoscale crystallites were prepared from aqueous solutions and gels containing CeCl_3_ and biological polymers such as gelatin and agar, by the addition of (NH_4_)_2_CO_3_ solutions. They were then thermally converted to CeO_2_ particles with the same morphologies [26]. Spherical SnO particles consisting of radially branched platy units were produced by ageing Sn_6_O_4_(OH)_4_ in aqueous solutions containing gelatin at 60°C [27]. Hence, biomimetic aqueous routes are promising ways to fabricate nanostructured inorganic materials.

In this work, nanostructured WO_3_ particles were prepared by a biomimetic aqueous solution process with gelatin. Tungsten oxides can exist in aqueous solutions as monomeric tungstate ions $\left(WO_{4}{}^{2-}\right)$ or para-tungstate ions ($HW_{6}O_{21}{}^{5-}$, $H_{2}W_{12}O_{42}{}^{10-}$, etc.) [28–32]. The ions can be precipitated as hydrous tungsten oxides (WO_3_ · *x*H_2_O) [33,34], tungstate (H_2_WO_4_, H_4_WO_5_, etc.) [35,36] crystals or WO_3_. The crystal phase and morphology of the precipitates are affected by the pH and by the concentrations of the precursors. Here, nanostructured WO_3_ · H_2_O particles were prepared as WO_3_ precursors from (NH_4_)_10_W_12_O_41_ · 5H_2_O aqueous solutions that contained gelatin; the WO_3_ particles were subsequently obtained by heating the precursors. As mentioned above, we have previously tried to prepare nanostructured CeO_2_ materials on the basis of a similar strategy [26]. In that work, nanostructured CeCO_3_OH particles were obtained with biological polymers and then thermally converted to CeO_2_, while the crystallographic orientation of inorganic units like biominerals was not observed in the CeO_2_ products [26]. On the other hand, WO_3_ · H_2_O were reported to topotactically transform to monoclinic WO_3_ crystals [33], which would allow us to keep the crystallographic orientation of inorganic units after the thermal conversion to metal oxide. We varied the pH and the gelatin concentration to investigate the effects on size, shape and crystal phase of the WO_3_ precursors and WO_3_ particles.

## 2. Material and methods

Aqueous HCl solutions with pH 0.6–1.0 were prepared by diluting 36.0 mass % hydrochloric acid (Wako Pure Chemical Industries, Osaka, Japan) with purified water. Then, 0.10 g of (NH_4_)_10_W_12_O_41_ · 5H_2_O (Wako Pure Chemical Industries, Osaka, Japan) was dissolved in 20 cm^3^ of the HCl solutions by stirring at 80°C for 3 min. When 0–0.040 g of gelatin (Wako Pure Chemical Industries, Osaka, Japan) was added, the solutions immediately became cloudy. After stirring at 80°C for 3 h, the cloudy suspensions became transparent and were then used as the precursor solutions ([(NH_4_)_10_W_12_O_41_ · 5H_2_O] = 1.7 mM, [gelatin] (*C*_ge_) = 0–2.0 g l^−1^). These solutions were aged at 60°C for 1–7 days, resulting in yellowish precipitates that were washed with purified water and dried at 60°C for 24 h. The precipitates were WO_3_ precursors that were heated at a rate of 5°C min^−1^ to 600°C, which was maintained at the heating temperature (600°C) for 24 h in the air for conversion to WO_3_.

The crystalline phases of the WO_3_ precursors and the heat-treated WO_3_ products were identified by X-ray diffraction (XRD) in an ordinary 2*θ*/*θ* mode, with a CuK*α* X-ray diffractometer (Model Rint 2550V, Rigaku, Tokyo, Japan) operated at 40 kV and 300 mA. The microstructures of the precursors and the heat-treated samples were imaged with a field-emission scanning electron microscope (FE-SEM) (Model JSM-6500F, JEOL, Tokyo, Japan) and a field-emission transmission electron microscope (FE-TEM) (JEM-2000EX, JEOL, Tokyo, Japan). Thermogravimetric and differential thermal analysis (TG–DTA) curves were obtained for the WO_3_ precursors at a heating rate of 10°C min^−1^ in flowing air with a thermal analyser (Model ThermoPlus 2, Rigaku, Tokyo, Japan).

## 3. Results and discussion

### 3.1. Preparation of WO_3_ precursors

At first, we performed preliminary experiments to know the reaction time at which the increase in the sample yield stopped. Aqueous solutions of 1.7 mM [(NH_4_)_10_W_12_O_41_ · 5H_2_O] and 0–2.0 g l^−1^ gelatin (*C*_ge_) with HCl at pH 0.6–1.0 were aged at 60°C for 1–7 days. Yellowish WO_3_ precursors were precipitated by ageing irrespective of *C*_ge_ and pH. The ageing times at which the increase in the sample yield stopped, and precursor yields are listed in table 1.

**Table 1.** Ageing times and precursor yields.

pH of solvents	[gelatin] (g l^−1^)	ageing time (day)	yield (%)
0.6	0	1.0	63.3
0.6	0.1	1.0	30.2
0.6	0.2	1.1	24.0
0.6	0.5	3.6	28.0
0.6	1.0	5.8	31.3
0.6	1.5	5.0	25.7
0.6	2.0	7.0	30.0
0.8	0	1.0	47.2
0.8	0.1	1.1	33.2
0.8	0.2	2.0	25.6
0.8	0.5	5.0	18.6
0.8	1.0	5.0	39.3
0.8	1.5	5.7	26.1
0.8	2.0	5.0	21.2
1.0	0	1.0	55.2
1.0	0.1	4.0	25.4
1.0	0.2	4.0	23.4
1.0	0.5	5.0	39.5
1.0	1.0	7.0	22.0
1.0	1.5	7.0	22.3
1.0	2.0	7.0	18.3

**Figure 1.**
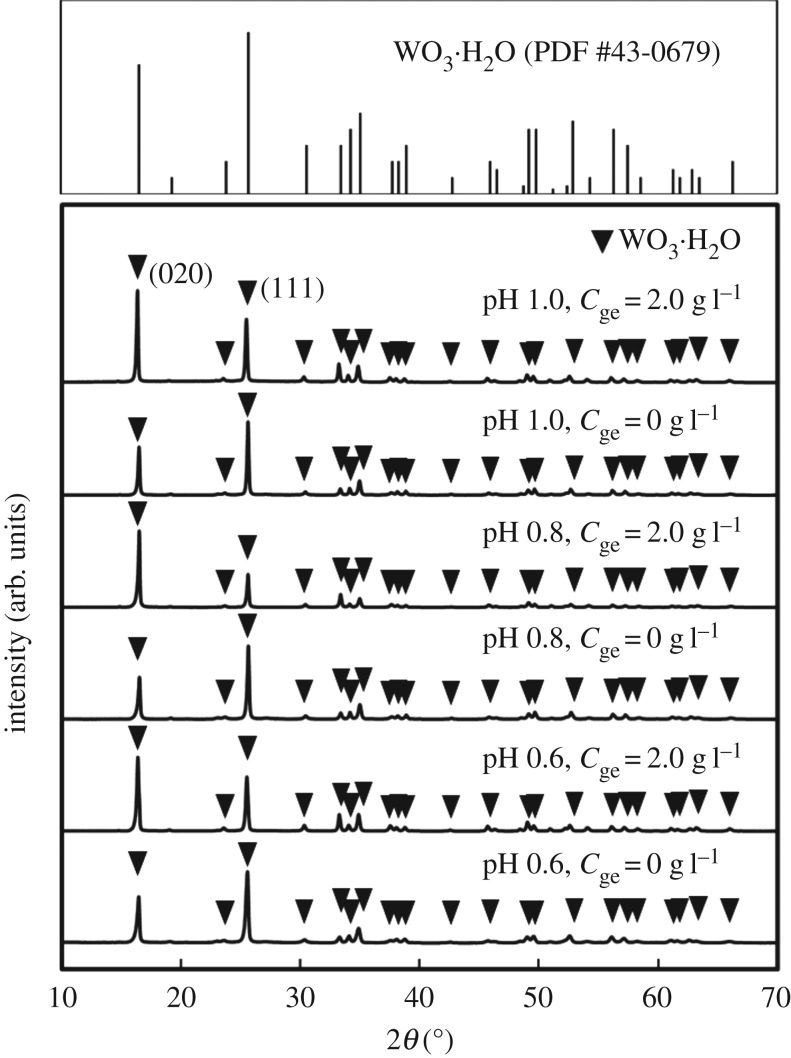
XRD patterns of WO_3_ precursors prepared from (NH_4_)_10_W_12_O_41_ solutions with *C*_ge_ = 0–2.0 g l^−1^ and HCl at pH 0.6–1.0 (the ageing time was as shown in table 1).

The precipitation of the WO_3_ precursors was slower and the yield decreased with increasing pH, which indicated that nucleation was suppressed by the decreased acidity. Tungsten oxides precipitate as hydrous tungstic acid (H_2_WO_4_ · *n*H_2_O) [35,36] and tungsten trioxide (WO_3_ · *n*H_2_O) [33,34] under strongly acidic conditions, and their solubility increases with pH [28–32]. In the present case, the higher solubility under more weakly acidic conditions caused a slower nucleation rate and thus a lower yield of WO_3_ precursors. Moreover, the addition of gelatin also inhibited the deposition of WO_3_ precursors because its amino groups might have coordinated with tungstate ions, leading to suppressed nucleation. On the basis of these results, we employed the ageing time described in table 1 for sample preparation. Figure 1 shows XRD patterns of the WO_3_ precursors. The diffraction peaks attributed to WO_3_ · H_2_O were observed irrespective of the pH and the gelatin concentration in the precursor solutions. The peak intensities of the (020) plane of the precursors prepared with gelatin were higher than those in the powder diffraction file (WO_3_ · H_2_O: PDF#43-0679). The precipitation of WO_3_ · H_2_O under an acidic condition seems to be as follows:

3.1 $$WO_{4}{}^{2-}+2H^{+}\to WO_{3}\cdot H_{2}O.$$

This reaction consumes H^+^ ions, resulting in the increase in the pH value. On the other hand, in the present case, the pH value is almost unchanged after the precipitation. Here, the [(NH_4_)_10_W_12_O_41_ · 5H_2_O] was very low (1.7 mM), and thus the pH change was deduced to be small during the reaction.

**Figure 2.**
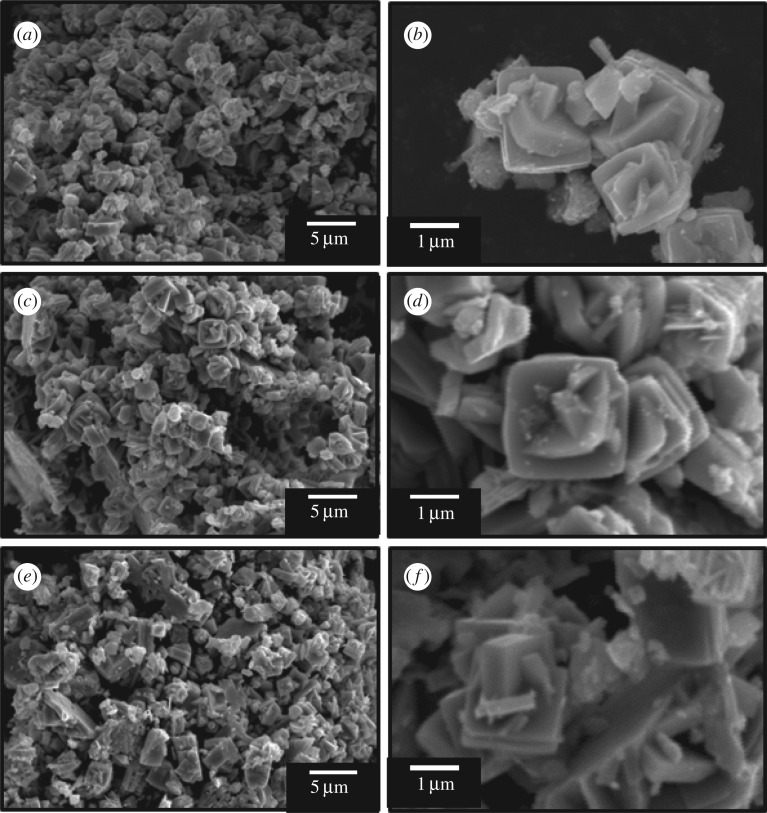
SEM images of WO_3_ precursors prepared from (NH_4_)_10_W_12_O_41_ solutions with *C*_ge_ = 0 g l^−1^ and HCl at pH 0.6 (*a*,*b*), pH 0.8 (*c*,*d*) and pH 1.0 (*e*,*f*) (the ageing time was as shown in table 1).

**Figure 3.**
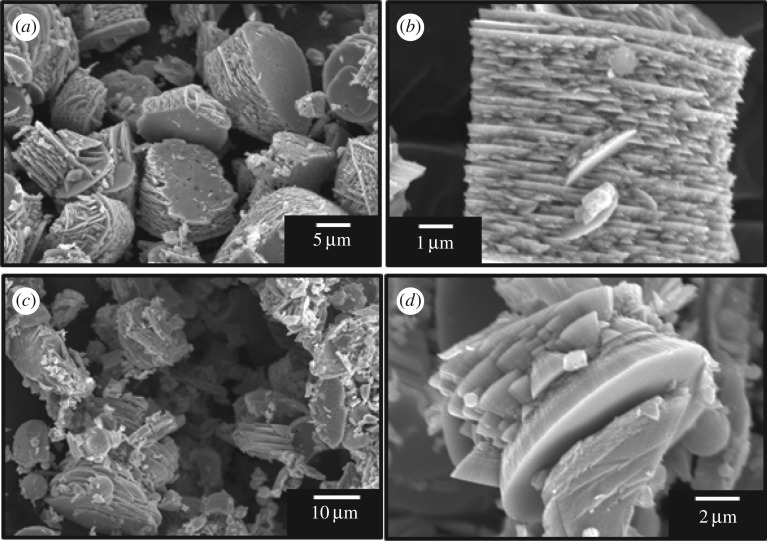
SEM images of WO_3_ precursors prepared from (NH_4_)_10_W_12_O_41_ solutions with *C*_ge_ = 0.2 g l^−1^ (*a*,*b*) and 2.0 g l^−1^ (*c*,*d*) and HCl at pH 0.6 (the ageing time was as shown in table 1).

**Figure 4.**
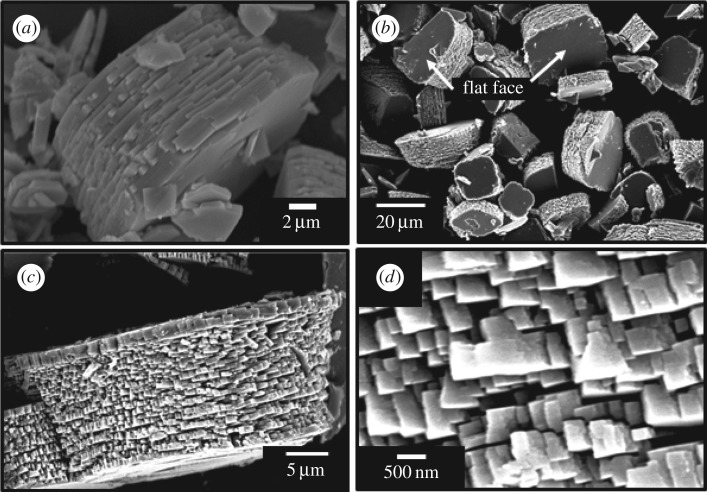
SEM images of WO_3_ precursors prepared from (NH_4_)_10_W_12_O_41_ solutions with *C*_ge_ = 0.2 g l^−1^ (*a*) and 1.5 g l^−1^ (*b*–*d*) and HCl at pH 1.0 (the ageing time was as shown in table 1).

Figure 2 shows SEM images of WO_3_ precursors prepared from (NH_4_)_10_W_12_O_41_ · 5H_2_O solutions with HCl at pH 0.6–1.0 without gelatin (*C*_ge_ = 0 g l^−1^). Random aggregates of platy particles 1–2 µm in width were obtained irrespective of the pH. The morphology of the WO_3_ precursors changed with the addition of gelatin, and its effect varied with the pH. SEM microstructure images of WO_3_ precursors prepared with gelatin (*C*_ge_ = 0–2.0 g l^−1^) are shown in figures 3 and 4, for (NH_4_)_10_W_12_O_41_ · 5H_2_O solutions with HCl at pH 0.6 and 1.0, respectively. Under relatively strong acidic conditions (pH 0.6), layered structures with widths of 10 µm and thicknesses of 5–10 µm appeared when *C*_ge_ = 0.2 g l^−1^ (figure 3*a,b*); the structures consisted of stacked platy units. For *C*_ge_ > 0.5 g l^−1^, the plate-like microstructure collapsed, resulting in unshaped aggregates with inhomogeneous sizes and shapes (figure 3*c*,*d*). Under weakly acidic conditions (pH 1.0), layered structures were obtained by the addition of gelatin (figure 4*a*), as well as more acidic conditions (pH 0.6) (figure 3*a*,*b*). In weakly acidic conditions, the addition of large amounts of gelatin did not result in the collapse of the platy structure. The increase to *C*_ge_ = 1.5 g l^−1^ induced the formation of large layered plates with widths of 20–50 µm and thicknesses of 10–20 µm (figure 4*b*,*c*), with relatively homogeneous sizes and shapes. Moreover, segmented block-like units of 0.50–1 µm in size were observed on the side faces of the plates (figure 4*d*), which suggested that large amounts of gelatin caused branching of the platy particles.

**Figure 5.**
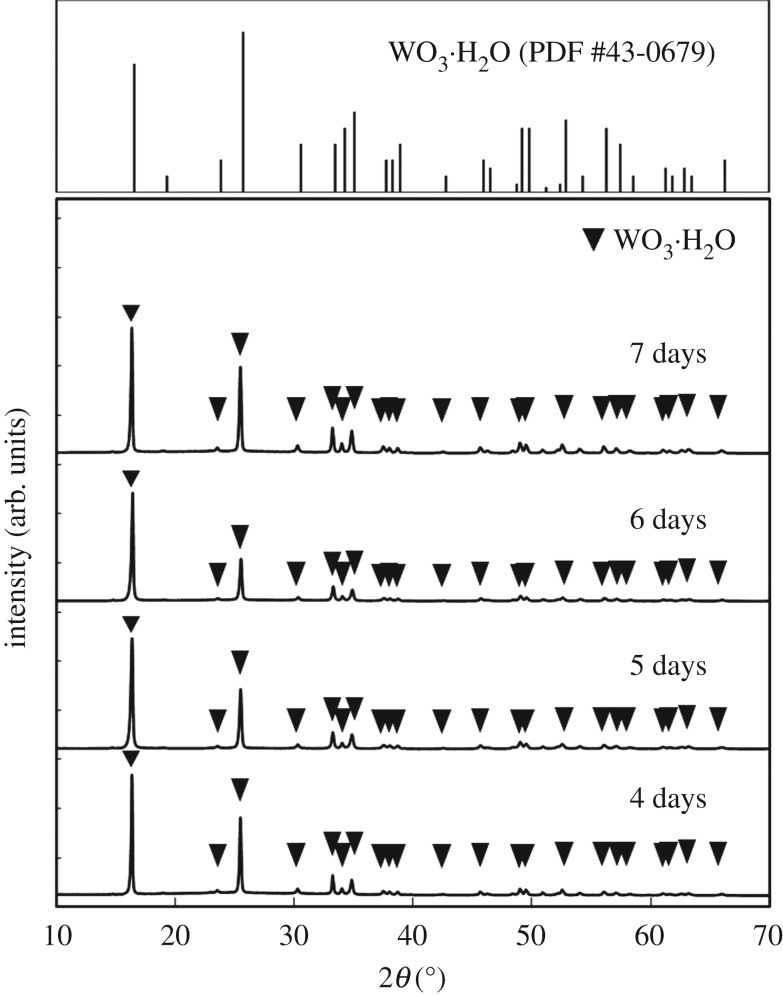
XRD patterns of WO_3_ precursors prepared by ageing for 4–7 days from (NH_4_)_10_W_12_O_41_ solutions with *C*_ge_ = 2.0 g l^−1^ and HCl at pH 1.0.

**Figure 6.**
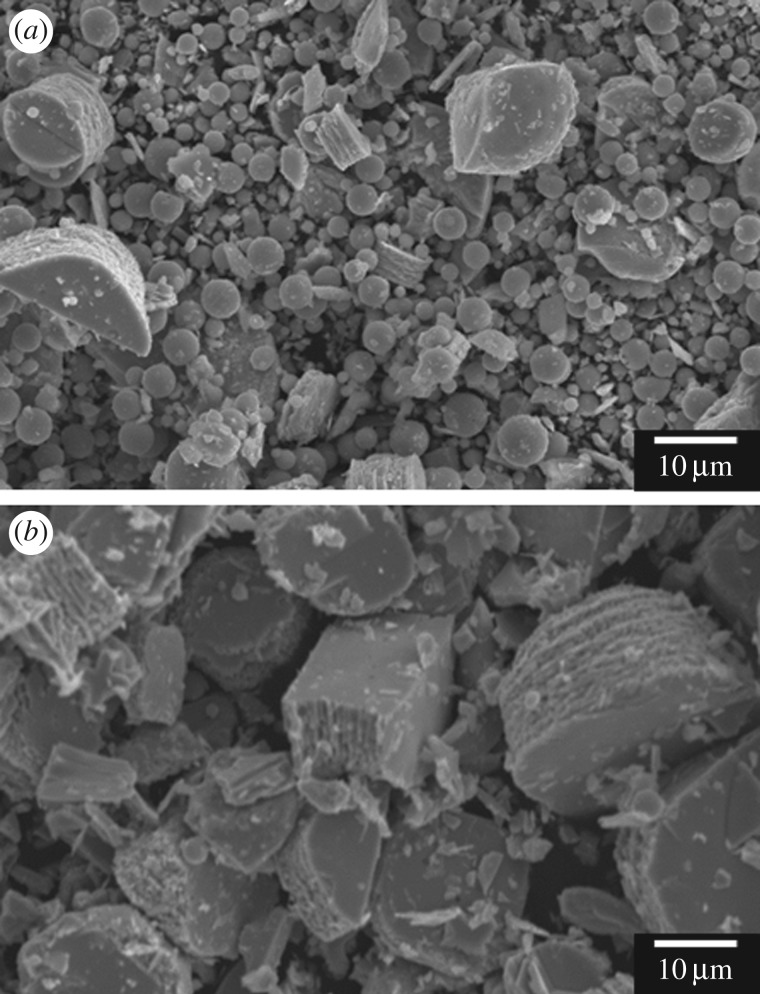
SEM images of WO_3_ precursors prepared by ageing for 4 (*a*) and 6 (*b*) days from (NH_4_)_10_W_12_O_41_ solutions with *C*_ge_ = 2.0 g l^−1^ and HCl at pH 1.0.

We investigated the effect of the ageing time on the morphology and crystal phase of WO_3_ precursors. WO_3_ precursors were prepared by ageing for 1–7 days from (NH_4_)_10_W_12_O_41_ solutions with *C*_ge_ = 2.0 g l^−1^ and HCl at pH 1.0. No precipitation was observed for 1–3 days, while precipitates appeared after 4 days. Figure 5 shows the XRD patterns of WO_3_ precursors prepared by ageing for 4–7 days. The diffraction peaks attributed to WO_3_ · H_2_O were observed irrespective of the ageing times. Figure 6 shows the SEM images of the WO_3_ precursors. The precipitate obtained on 4 days was the mixture of layered plates and spherical particles (figure 6*a*). The spherical particles disappeared with increasing ageing times, and only layered plates were observed after 6 days (figure 6*b*). The spherical particles found in the precipitates of 4–5 days were thought to be the composites of tungstate ions and gelatin. As described in the experimental section, in this work, the (NH_4_)_10_W_12_O_41_ aqueous solutions immediately became cloudy on addition of gelatin, which might be attributed to the formation of the composites of tungstate ions and gelatin. The cloudy suspension became transparent again by stirring at 80°C and then was used as the precursor solutions. In the case of the 4–5 days ageing, the precipitation of WO_3_ · H_2_O did not complete, and thus unreacted tungstate ions remained in the solutions. The tungstate ions might precipitate as the gelatin composite during cooling, forming the spherical particles.

In the present work, the addition of gelatin led to the formation of WO_3_ · H_2_O layered structures (figures 3 and 4). The WO_3_ precursors prepared with gelatin exhibited an intense diffraction peak from the (020) plane (figure 1), which indicated that the flat face of the layered structures was the (010) plane of WO_3_ · H_2_O crystals. This agreed well with previous reports that the synthesis of WO_3_ · H_2_O plates exposed the (010) plane as the flat face [7,33,37]. The morphological change of the WO_3_ precursors from random aggregates of platy particles (figure 2) to layered structures (figures 3 and 4) was attributed to the adsorption of gelatin on the (010) planes of the WO_3_ · H_2_O crystals. Without gelatin, heterogeneous nucleation rapidly occurred on the surface of WO_3_ · H_2_O platy particles and crystallites grew in various directions, resulting in random aggregates (figure 2). Alternatively, gelatin might have adsorbed on the (010) plane of the WO_3_ · H_2_O crystals and suppressed nucleation and crystal growth on the surface of the plates. The mild nucleation and growth rates could have caused the slow growth of new platy crystallites along the flat face of the WO_3_ · H_2_O plates, resulting in layered structures (figure 3*a*,*b* and figure 4).

Moreover, weakly acidic conditions (pH 1.0) resulted in large layered plates with more homogeneous sizes and shapes (figure 4*b*). This could have been caused by the slower nucleation rate because of the higher solubility of the tungsten compounds. In such a case, the branching growth of platy particles occurred with increasing gelatin concentration (figure 4*d*). The larger amounts of gelatin could adsorb on the side face of WO_3_ · H_2_O plates as well as the flat face, which inhibited the growth in the lateral direction. This resulted in the branching of platy particles and the subsequent formation of the segmented block-like units (figure 4*d*).

### 3.2. Thermal conversion to WO_3_ particles

**Figure 7.**
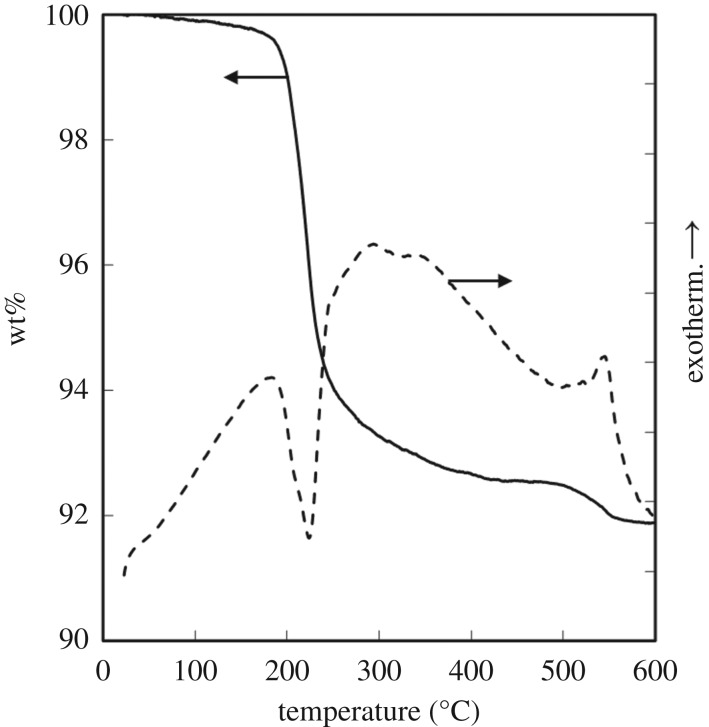
TG–DTA curves for WO_3_ precursors prepared from (NH_4_)_10_W_12_O_41_ solutions with *C*_ge_ = 2.0 g l^−1^ and HCl at pH 1.0 (the ageing time was as shown in table 1).

The WO_3_ precursors (WO_3_ · H_2_O) obtained with gelatin were heated for thermal conversion to WO_3_. Figure 7 shows TG–DTA curves for the WO_3_ precursors (*C*_ge_ = 2.0 g l^−1^, pH 1.0). The first weight loss with an endothermic peak at 220°C indicated dehydration and was close to the theoretical weight loss (7.2 wt%) of the reaction

3.2 $$WO_{3}\cdot H_{2}O\to WO_{3}+H_{2}O.$$

**Figure 8.**
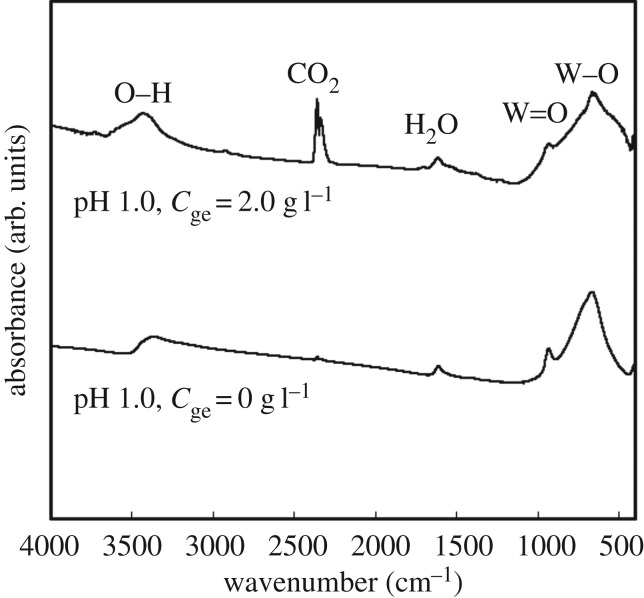
FT-IR spectra for WO_3_ precursors prepared from (NH_4_)_10_W_12_O_41_ solutions with *C*_ge_ = 0 and 2.0 g l^−1^ and HCl at pH 1.0 (the ageing time was as shown in table 1).

In addition, a slight weight loss of 1 wt% was detected at 550°C, which could be attributed to the combustion of residual gelatin. We also investigated the presence of residual gelatin on the WO_3_ precursors by FT-IR analysis. Figure 8 shows FT-IR spectra for the WO_3_ precursors (*C*_ge_ = 0 and 2.0 g l^−1^, pH 1.0). No absorption peaks due to gelatin were detected, which also indicates that only a little amount of gelatin remained on the WO_3_ · H_2_O products.

**Figure 9.**
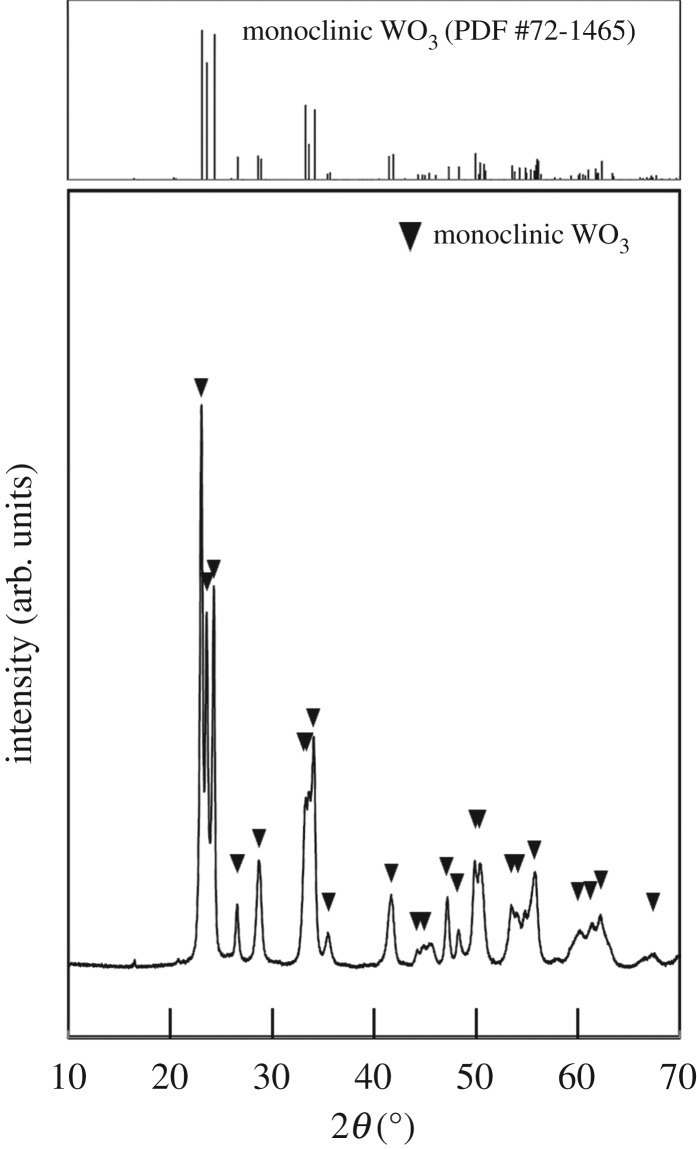
XRD patterns of heat-treated WO_3_ products obtained from WO_3_ precursors prepared by ageing for 7 days from (NH_4_)_10_W_12_O_41_ solutions with *C*_ge_ = 1.5 g l^−1^ and HCl at pH 1.0.

The large WO_3_ · H_2_O layered plates (*C*_ge_ = 1.5 g l^−1^, pH 1.0) were converted to WO_3_ by heat treatment at 600°C for 24 h in air. Figure 9 shows the XRD patterns of the heat-treated WO_3_. Diffraction peaks attributed to monoclinic WO_3_ were observed, and the WO_3_ · H_2_O phase was absent.

**Figure 10.**
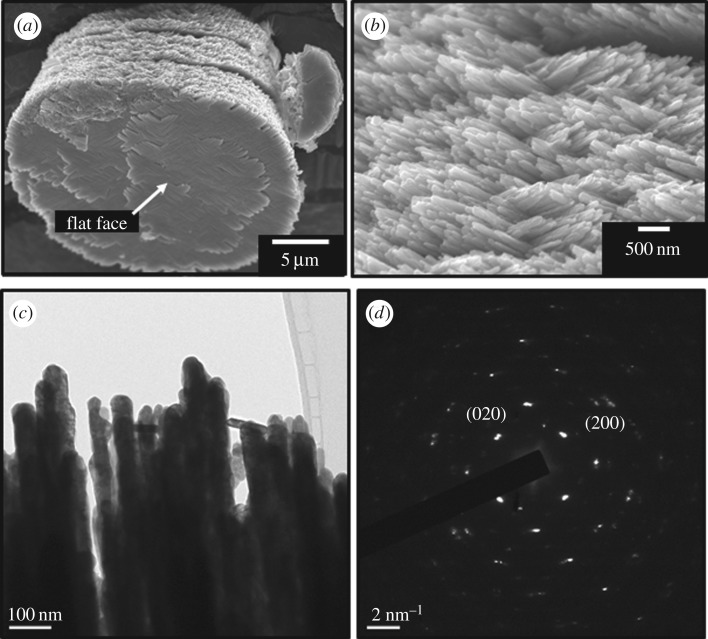
FE-SEM (*a*,*b*) and FE-TEM (*c*,*d*) images of heat-treated WO_3_ products from WO_3_ precursors prepared by ageing for 7 days from (NH_4_)_10_W_12_O_41_ solutions with *C*_ge_ = 1.5 g l^−1^ and HCl at pH 1.0.

Figure 10 shows FE-SEM and FE-TEM images of the heat-treated WO_3_ products (*C*_ge_ = 1.5 g l^−1^, pH 1.0). As shown in figure 10*a*, the layered structure of the WO_3_ precursors remained after thermal conversion to WO_3_. In addition, orthogonally crossed nanorods 50 nm in width were observed in the WO_3_ layers (figures 10*b*,*c*). Regular diffraction spots were observed in the selected area electron diffraction pattern of the WO_3_ (figure 10*d*). The diffraction spots indicated that the rod-like units were oriented in the same crystallographic direction, and the flat face of heat-treated WO_3_ layers is (001) plane of monoclinic WO_3_. WO_3_ · H_2_O were reported to topotactically transform to monoclinic WO_3_ crystals [33]. As discussed in the XRD measurement of WO_3_ precursors, the flat face of the WO_3_ · H_2_O layered structures was the (010) plane of WO_3_ · H_2_O crystals. These suggest the topotactic transformation of [010]-oriented WO_3_ · H_2_O layered plates to [001]-oriented WO_3_. These results suggested that the WO_3_ layered structures prepared with gelatin have highly ordered nanostructures consisting of oriented inorganic nanoscale units like biominerals such as nacres, sea urchin spines and eggshells [9–17]. We evaluated the BET surface area of the WO_3_ products by N_2_ adsorption method. The surface area of the layered structure obtained by an addition of gelatin (*C*_ge_ = 1.5 g l^−1^, pH 1.0) was 5.02 m^2^ g^−1^, which was larger than that of the random aggregates of *C*_ge_ = 0 g l^−1^, pH 1.0 (2.31 m^2^ g^−1^). The hierarchical nanostructures are thought to be suitable for photoelectrode and gas sensor materials.

## 4. Conclusion

WO_3_ particles with highly ordered nanostructures were prepared via a biomimetic process involving the biological polymer gelatin. WO_3_ · H_2_O platy particles were WO_3_ precursors obtained from (NH_4_)_10_W_12_O_41_ · 5H_2_O aqueous solutions, where the addition of gelatin resulted in morphological changes from random aggregates to layered structures. The layered structures consisted of platy particles branching to block-like nanoscale units that were induced by the suppression of nucleation and growth by gelatin adsorption. Nanostructured WO_3_ particles were obtained from the WO_3_ · H_2_O layered structures by heat treatment. A morphological change of nanoscale units from segmented blocks to orthogonally crossed nanorods was observed. Overall, these results suggested that an aqueous route mimicking biomineralization was effective for nanostructural control of inorganic materials.

## Supplementary Material

Reviewer comments

## Data accessibility

There are no additional data to accompany this manuscript. All relevant data (the table of ageing times and product yields, XRD patterns, SEM and TEM images and TG–DTA curves) are within the main body of the manuscript.
